# Predictors of transfer from a remote trauma facility to an urban level I trauma center for blunt splenic injuries: a retrospective observational multicenter study

**DOI:** 10.1186/s13037-022-00339-4

**Published:** 2022-09-09

**Authors:** Constance McGraw, Charles W. Mains, Jodie Taylor, Cecile D’Huyvetter, Kristin Salottolo, David Bar-Or

**Affiliations:** 1 Injury Outcomes Network, Englewood, CO USA; 2 Trauma Services Department, Centura Health Trauma System, Centennial, CO USA; 3 Trauma Services Department, St. Anthony Summit Hospital, Frisco, CO USA

**Keywords:** Transfers, Spleen, Observation, Triage

## Abstract

**Background:**

The decision-making for admission versus emergent transfer of patients with blunt splenic injuries presenting to remote trauma centers with limited resources remains a challenge. Although splenectomy is standard for hemodynamically unstable patients, the specific criterion for non-operative management continues to be debated. Often, lower-level trauma centers do not have interventional radiology capabilities for splenic artery embolization, leading to transfer to a higher level of a care. Thus, the aim of this study was to identify specific characteristics of patients with blunt splenic injuries used for admittance or transfer at a remote trauma center.

**Methods:**

A retrospective observational study was performed to examine the management of splenic injuries at a mountainous and remote Level III trauma center. Trauma patients ≥ 18 years who had a blunt splenic injury and initially received care at a Level III trauma center prior to admittance or transfer were included. Data were collected over 4.5 years (January 1, 2016 – June 1, 2020). Patients who were transferred out in > 24 h were excluded. Patient demographics, injury severity, spleen radiology findings, and clinical characteristics were compared by decision to admit or transfer to a higher level of care ≤ 24 h of injury. Results were analyzed using chi-square, Fisher’s exact, or Wilcoxon tests. Multivariable logistic models were used to identify predictors of transfer.

**Results:**

Of the 73 patients included with a blunt splenic injury, 48% were admitted and 52% were transferred to a Level I facility. Most patients were male (*n* = 58), were a median age of 26 (21–42) years old, most (*n* = 62) had no comorbidities, and 47 had been injured from a ski/snowboarding accident. Compared to admitted patients, transferred patients were significantly more likely to be female (13/38 vs. 3/36, *p* = 0.007), to have an abbreviated injury scale score ≥ 3 of the chest (31/38 vs. 7/35, *p* = 0.002), have a higher injury severity score (16 (16–22) vs. 13 (9–16), *p* = 0.008), and a splenic injury grade ≥ 3 (32/38 vs. 12/35, *p* < 0.001). After adjustment, splenic injury grade ≥ 3 was the only predictor of transfer (OR: 12.1, 95% CI: 3.9–37.3, *p* < 0.001). Of the 32 transfers with grades 3–5, 16 were observed, and 16 had an intervention. Compared to patients who were observed after transfer, significantly more who received an intervention had a blush on CT (1/16 vs. 7/16, *p* = 0.02) and a higher median spleen grade of 4 (3–5) vs. 3 (3–3.5), *p* = 0.01).

**Conclusions:**

Our data suggest that most patients transferred from a remote facility had a splenic injury grade ≥ 3, with concomitant injuries but were hemodynamically stable and were successfully managed non-operatively. Stratifying by spleen grade may assist remote trauma centers with refining transfer criteria for solid organ injuries.

**Supplementary Information:**

The online version contains supplementary material available at 10.1186/s13037-022-00339-4.

## Background

Blunt splenic injury is a leading cause of hemorrhage, often resulting in shock and mortality. Splenic injury accounts for 31% to 50% of blunt abdominal trauma [1]. Historically patients with splenic injury were surgically managed, with removal of the injured spleen [2]; however, the inception of high-resolution diagnostic imaging and angiography has changed the paradigm in management of splenic injuries towards nonoperative management (NOM) due to the enhanced accuracy of determining the severity of injury as well as the ability to treat [2, 3]. NOM includes angiography and embolization (SAE), which consists of identifying the vascular injury and/or hemorrhage through angiography, followed by embolization to control bleeding. Though observational management is the standard for low grade splenic injuries (≤ 3), there is no preferred treatment for splenic injury grades > 3 and it frequently varies by trauma center level and location [4].

Current guidelines and recommendations by the Eastern Association for the Surgery of Trauma (EAST) 2016 [5] support the following: operative management (OM) is an absolute indication for patients with hemodynamic instability who do not respond to initial resuscitation. NOM with observation is reserved for stable grade I-III blunt splenic injury, a repeat CT should be considered in grade III patients with a contrast blush to determine need for NOM-SAE, and grade IV/V injury without hypotension or a large hemoperitoneum should also be considered for NOM-SAE. However, particularly for trauma centers in remote areas where time to treatment is crucial, SAE is frequently unavailable or can be a time-consuming specialized procedure, requiring an interventional radiologist that may not be emergently available. Thus, lower-level trauma centers with limited resources must expeditiously decide who can be immediately transferred to a higher level of care for SAE and who can be safely admitted for either surgery (exploratory laparotomy and splenectomy) or observation. Although there are a proliferation of studies examining the triage of splenic injuries [3, 6–15], there are a paucity of studies on management of patients with blunt splenic injuries in remote settings and of the few that exist, most report very small numbers and are not current [16, 17]. There continues to be great variability in the processes of care for splenic injury patients, with sparse data reported across Level III or IV trauma centers.

Across inclusive trauma systems, integrated networks of trauma centers are streamlined for referrals for definitive care across vast geography and may help improve access to care and outcomes; however, these hospitals may not be uninformedly distributed across states [18, 19]. Our Level III trauma center is located in the mountains with an elevation of around 9,000 feet [20], there are over 100 days/year of being “transport challenged” due to weather, and the closest Level I center is around 75 miles by ground. Thus, the decision to admit or transfer must take many additional factors into account compared to urban facilities. Because there is no IR service at this Level III facility, transfer agreements were developed requiring all patients with high risk of needing SAE be transferred. Typically, transfers are completed in hemodynamically stable patients with grade 4 and 5 splenic injuries.

The study objective was to identify a common set of clinical characteristics used for admittance or transfer, as well as to describe management and outcomes at each step of the way, which may eventually assist with defining and refining the transfer criteria. We hypothesized that this Level III trauma center may be admitting more patients due to inclement weather leading to a higher rate of splenectomies, that may have otherwise been transferred and have undergone appropriate NOM at the receiving facility.

## Methods

This retrospective observational study included trauma patients ≥ 18 years who suffered from a blunt splenic injury and initially received care at a remote Level III trauma center in a mountainous region, and then were either admitted or transferred to a Level I trauma center. Patients were included over 4.5 years (January 1, 2016-June 1, 2020) and splenic injury was identified by the hospital trauma registry as International Classification of Diseases, ICD-10 diagnosis code of S36.0. Patient demographics, injury severity, spleen radiology findings, and clinical characteristics were compared by decision to admit or transfer to a higher level of care ≤ 24 h of injury. Patients who were dead on arrival, died in the emergency department before diagnostic work-up, and whose findings on initial assessment were not documented were excluded. According to the advanced trauma life support (ATLS) guidelines, hemodynamically unstable patients with a positive focused assessment with sonography (FAST) exam are admitted for emergent damage control laparotomy and potential splenectomy. Hemodynamically stable patients can be selected for nonoperative management, which includes observation and continuous monitoring of vitals, or transfer to a higher level of care for angiographic embolization. This study was approved by institutional review boards at the participating center (CommonSpirit Health Research Institute IRB # 1,661,269).

The following covariates were collected on each patient from the trauma registry: sex, age (≥ 18), race, injury severity score (ISS, 1–15, ≥ 16), hospital length of stay (LOS), ICU LOS, mechanism of injury (bike, fall, motor vehicle crash (MVC), ski/snowboard, other), admission Glasgow Coma Scale (GCS, 3–8, 9–13, 14–15), abbreviated injury severity (AIS,) score (≤ 3 vs. > 3) in-hospital mortality, and the existing comorbid conditions in this patient population: chronic obstructive pulmonary disease (COPD), hypertension, and smoker.

The following covariates were collected from patient electronic medical records: initial and final splenic injury grade (uses the American Association for the Surgery of Trauma Spleen Injury Scale, which is currently the most widely used grading system for splenic trauma and is classified into grades I-V using CT findings; 2018 version used) [21], presence of contrast blush and size of blush (small, moderate, large) in radiology imaging, hemoperitoneum quantified from CT findings and size (small, moderate, large). Additional variables that were abstracted from the electronic medical record included prehospital vitals (systolic blood pressure, diastolic blood pressure, heart rate, respiration rate levels during the first 24 h (g/dL), hemodynamic instability (defined as < 90 mm Hg), total units of blood products received, initial intervention technique, and definitive intervention technique.

The primary outcome variable was transfer status. Categorical variables were analyzed with χ^2^ tests and Fisher’s exact tests, and continuous data were analyzed using Wilcoxon Mann–Whitney U tests and Kruskal Wallis tests, as necessary.

Stepwise multivariable logistic regression modeling was used to identify predictors of transfer and entry and exit criterion were set to *P* = 0.2 and *P* = 0.05, respectively. Models were further stratified by splenic injury grade ≥ 3. A significance level of α < 0.05 and SAS 9.4 (Cary, NC) were used to conduct all statistical analyses.

## Results

Of the 73 patients with a blunt splenic injury, 35 (48%) were admitted and 38 (52%) were transferred to a Level I facility. Overall, most patients were male (*n* = 58), were a median age of 26 (21–42) years old, most (*n* = 62) had no comorbidities, and 47 had been injured from a ski/snowboarding accident. Compared to admitted patients, significantly more females were transferred, (13/38 vs. 3/36, *p* = 0.007, Table 1), more had an abbreviated injury scale score (AIS) ≥ 3 (vs < 3) of the chest (31/38 vs. 7/35, *p* = 0.002) and had a higher injury severity score (16 (16–22) vs. 13 (9–16), *p* = 0.008), in comparison to admitted patients.

**Table 1:** Overall characteristics by decision to admit or transfer

Variable, n (%)	Admitted, *N* = 35 (48%)	Transferred, *N* = 38 (52%)	*p*-value
Median age, years	28 (22–51)	24.5 (21–32)	0.07
Gender			**0.007**
* Female*	3 (8%)	13 (34%)	
* Male*	33 (92%)	25 (66%)	
Cause of injury			0.34
* Bike*	1 (3%)	4 (11%)	
* Fall*	4 (11%)	1 (3%)	
* MVC*	5 (14%)	9 (24%)	
* Ski/Snowboard*	24 (69%)	23 (61%)	
* Other*	1 (3%)	1 (3%)	
Comorbidities			0.23
* Hypertension*	3 (9%)	0 (0%)	
* COPD*	1 (3%)	0 (0%)	
* Smoker*	3 (9%)	4 (11%)	
* None*	28 (80%)	34 (89%)	
Admission GCS			0.24
* 14 to 15*	35 (100%)	34 (89%)	
* 9 to 13*	0 (0%)	1 (3%)	
* 3 to 8*	0 (0%)	3 (8%)	
Extra-abdominal injuries AIS ≥ 3 (vs. < 3)			
* Head or neck*	15 (71%)	14 (88%)	0.42
* Chest*	18 (51%)	31 (86%)	**0.002**
Median (IQR) ISS	13 (9–16)	16 (16–22)	**0.008**
Transport mode			0.60
* Ground*	24 (69%)	28 (74%)	
* Private*	11 (31%)	9 (24%)	
* Helicopter*	0 (0%)	1 (3%)	

*MVC* motor vehicle crash, *COPD* chronic obstructive pulmonary disease, *GCS* Glasgow Coma Scale, *IQR* interquartile range, *RTS* Revised Trauma Score, *AIS* Abbreviated Injury Scale, *ISS* injury severity score, *LOS* length of stay, *ICU* intensive care unit. Bold *p*-values indicate statistical significance at *p* < 0.05

Table 2 presents clinical parameters of splenic injury by decision to admit or transfer. Transferred patients were significantly more likely to have a splenic injury grade ≥ 3 (32/38 vs. 12/35, *p* < 0.001), Table 2) and a hemoperitoneum on imaging (19/38 vs. 5/35, *p* = 0.002). Transferred patients also had a significantly longer hospital length of stay than patients who were admitted (5 (4–8) vs. 3 (2–4), *p* < 0.001). Though there were more transfer patients with a positive FAST exam finding, it did not reach statistical significance. There were no other significant differences by patient disposition.

**Table 2:** Clinical characteristics of splenic injury by decision to admit or transfer

Variable, n (%)	Admitted, *N* = 35 (48%)	Transferred, *N* = 38 (52%)	*p*-value
First Prehospital vital signs			
* Respiration rate*	18 (16–20)	18 (16–20)	0.15
* Heart rate*	88 (76–99)	83.5 (68–99)	0.37
* Systolic blood pressure*	125 (114–130)	126.5 (115–134)	0.75
* Diastolic blood pressure*	75.3 (12.5)	74.0 (14.8)	0.79
Last pre-hospital vital signs			
* Respiration rate*	18 (16–20)	18 (16–20)	0.42
* Heart rate*	86 (78–100)	84.5 (70–99)	0.59
* Systolic blood pressure*	124 (117–130)	125.5 (115–137)	0.29
* Diastolic blood pressure*	75.2 (11.7)	76.5 (65–85)	0.92
Hemoglobin arrival	14.4 (12.8–15.3)	13.8 (13.2–15.9)	0.30
Initial spleen grade			** < 0.001**
* 1–2*	24 (69%)	6 (16%)	
* 3–5*	11 (31%)	32 (84%)	
Final spleen grade			** < 0.001**
* 1–2*	23 (66%)	6 (16%)	
* 3–5*	12 (34%)	32 (84%)	
Hemodynamic instability prior to arrival	4 (11%)	1 (3%)	0.19
Hemodynamic instability on arrival	5 (15%)	5 (14%)	0.65
FAST exam results			0.07
* Positive*	17 (61%)	27 (82%)	
* Negative*	11 (39%)	6 (18%)	
CT findings			
* Pseudoaneurysm*	2 (6%)	0 (0%)	0.21
* Contrast blush*	4 (12%)	9 (24%)	0.19
* Blush size*			0.27
* Small*	3 (75%)	3 (33%)	
* Moderate*	1 (25%)	6 (67%)	
* Hemoperitoneum*	5 (15%)	19 (50%)	**0.002**
* Hemoperitoneum size*			0.14
* Small*	2 (40%)	13 (68%)	
* Moderate*	2 (40%)	6 (32%)	
* Large*	1 (20%)	0 (0%)	
Median (IQR) Hospital LOS, *days*	3 (2–4)	5 (4–8)	** < 0.001**
Median (IQR) ICU LOS, *days*	2.5 (2.0–3.0)	3 (2–5)	0.06

*CT* computed tomography, *FAST* focused assessment with sonography, *IQR* interquartile range, *LOS* length of stay, *ICU* intensive care unit. Bold *p*-values indicate statistical significance at *p* < 0.05

After adjustment, splenic injury grade ≥ 3 was the only predictor of transfer (OR:12.1, 95% CI: 3.9–37.3, *p* < 0.001). Table 3 stratifies findings by splenic injury grade. In the subset with grades ≥ 3, compared to admitted patients, significantly more transferred patients were likely to be females (10/32 vs. 0/11, *p* = 0.04, Table 3), nearly all had a head AIS score ≥ 3, had a slightly higher ISS range, and more had a small hemoperitoneum on imaging. Additionally, eight (26%) patients with a splenic injury grade ≥ 3 had a blush on CT, with no significant differences between groups.

**Table 3:** Splenic injury characteristics by disposition, stratified by splenic injury grade

Variable, n (%)	Grades 1–2, *N* = 30		Grades 3–5, *N* = 43	
	Admitted, *N* = 24 (80%)	Transferred, *N* = 6 (20%)	Admitted, *N* = 11 (26%)	Transferred, *N* = 32 (74%)
Age, years				
≤ *18–25*	8 (33%)	5 (83%)	6 (55%)	18 (56%)
≥ *26*	16 (64%)	1 (17%)	5 (45%)	14 (44%)
Gender				
* Female*	3 (13%)	3 (50%)	**0 (0%)**	**10 (31%)**
* Male*	21 (88%)	3 (50%)	**11 (100%)**	**22 (69%)**
Cause of injury				
* Bike*	1 (4%)	1 (17%)	0 (0%)	3 (9%)
* Fall*	3 (13%)	0 (0%)	1 (9%)	1 (3%)
* MVC*	5 (21%)	4 (67%)	0 (0%)	5 (16%)
* Ski/Snowboard*	14 (58%)	1 (17%)	10 (91%)	22 (69%)
* Other*	1 (4%)	0 (0%)	0 (0%)	1 (3%)
Comorbidities				
* Hypertension*	2 (8%)	0 (0%)	1 (9%)	0 (0%)
* COPD*	0 (0%)	0 (0%)	1 (9%)	0 (0%)
* Smoker*	3 (12%)	1 (20%)	0 (0%)	3 (9%)
* None*	19 (79%)	5 (83%)	9 (82%)	29 (91%)
Admission GCS				
* 14 to 15*	24 (100%)	4 (67%)	11 (100%)	30 (94%)
* 9 to 13*	0 (0%)	1 (17%)	0 (0%)	0 (0%)
* 3 to 8*	0 (0%)	1 (17%)	0 (0%)	2 (6%)
AIS ≥ 3 (vs. < 3)				
* Head or neck*	12 (75%)	2 (67%)	3 (60%)	12 (92%)
* Chest*	7 (29%)	1 (25%)	11 (100%)	30 (94%)
Median (IQR) ISS	13 (8–15)	15.5 (9–25)	16 (9–16)	16 (16–21.5)

*MVC* motor vehicle crash, *COPD* chronic obstructive pulmonary disease, *GCS* Glasgow Coma Scale, *AIS* abbreviated injury scale, *IQR* interquartile range, *ISS* injury severity score. Bold numbers indicate statistical significance at *p* < 0.05

Only one transferred patient with a splenic injury grade ≥ 3 experienced hemodynamic instability prior to arrival which subsided before decision to transfer, and five experienced hemodynamic instability upon arrival to the higher level of care (refer to supplementary Tables 1 & 2 for clinical characteristics by grade).

Table 4. outlines transfer patients by splenic injury grade 3–5 and management strategies. Ultimately, of the 38 transfers, 21 (55%) were observed, 14 (37%) had SAE, and three (7%) had surgery. Compared to patients who were transferred and were observed, patients who were transferred and had a procedure (SAE or splenectomy) had a significantly higher median splenic injury grade of 4 (3–4) (vs 3 (3–3.5) *p* = 0.01, Table 4), significantly more had a blush on CT (7/16 vs. 1/16, *p* = 0.02), of which most (*n* = 6) were moderately sized, and had a slightly higher ISS. One transfer patient with a splenic injury grade of 5 failed NOM.

**Table 4:** Characteristics of transfer patients with splenic injury grades 3–5 by management

Characteristics	Observation, *N* = 16 (50%)	Procedure, *N* = 16 (50%)	*p*-value
Gender			0.13
* Female*	3 (19%)	7 (44%)	
* Male*	13 (81%)	9 (56%)	
AIS regions and scores			
* Head* ≥ *3*	7 (100%)	5 (83%)	0.46
* Chest* ≥ *3*	14 (88%)	16 (100%)	0.48
FAST exam results	8 (67%)	15 (94%)	0.13
ISS, median (IQR)	16 (14.5–17.5)	17 (16–25)	0.29
Blush on CT	1 (7%)	7 (44%)	**0.02**
Blush size			0.25
* Small*	1 (100%)	1 (14%)	
* Moderate*	0 (0%)	6 (86%)	
Hemoperitoneum	8 (50%)	11 (69%)	0.28
Hemoperitoneum size			0.38
* Small*	4 (50%)	8 (73%)	
* Moderate*	4 (50%)	3 (27%)	
* Large*	0 (0%)	0 (0%)	
Spleen grade, median (IQR)	3 (3–3.5)	4 (3–4)	**0.01**

*AIS* abbreviated injury scale, *IQR* interquartile range, *CT* computed tomography; Bold *p*-values indicate statistical significance at *p* < 0.05

## Discussion

There has been a shift in recent years towards using SAE for patients suffering from splenic injuries to preserve function, as it has been shown to be safe and effective, as well as require less resources and time spent in the hospital. Lower-level trauma centers, however, frequently do not have SAE capabilities and must make clinical decisions prudently on triage strategies. Though multiple studies discuss splenic injury management, very few take place in a remote setting. This study described in detail, the predictors for transfer, management, and outcomes of patients with a blunt splenic injury who were triaged through a mountainous Level III trauma center. Overall, the main predictor of transfer to a higher level of care was having an splenic injury grade ≥ 3 and concomitant injuries, most had a small hemoperitoneum, and the majority were ultimately managed with NOM by observation upon admission to a higher level of care. Furthermore, no transfer patients planned for SAE were admitted to the Level III due to weather and inappropriately managed. Because the rate of failure of NOM was minimal, using spleen grade, hemodynamic status, and concomitant injuries under clinical supervision may help triage splenic injuries in this patient population.

Though there is considerable literature discussing management strategies of splenic injuries, to our knowledge, this is one of the first studies to specifically describe the patient population and triage strategy of patients with a blunt splenic injury who were injured in a remote area. Harwell et al. describe rural trauma patients who underwent damage control laparotomy (damage control laparotomy) prior to or following transfer, and showed that although damage control laparotomy is not a new procedure in urban trauma centers, it is still a relatively uncommon procedure in remote settings [22]. Similar to our study, they outlined clinical parameters of each group (damage control laparotomy at a rural facility, stable with damage control laparotomy after transfer, unstable with damage control laparotomy after transfer) and determined which patients can safely be transferred, and who should be admitted. In Harwell et al., unstable patients who were transferred and had immediate laparotomy in this study had more than double the ISS of the other groups, equating to multiple concomitant injuries including basilar skull fractures, liver lacerations, distal aortic lacerations, pneumothoraxes, and splenic injuries. We also found that concomitant injuries, including femoral artery dissections, pneumothoraxes, multiple rib fractures, hemothoraces, as well as findings on CT such as hemoperitoneum and contrast blush, determined need for transfer. Additionally, upon arrival to a higher-level trauma center, 45% of the transfer patients received some sort of intervention (SAE or surgery). Patients who received SAE tended to have rib fractures, blushes, hemoperitoneums, and pneumothoraces, while the three patients who had surgery all had an initial splenic grade of four, two had rib fractures, and one had a moderate hemoperitoneum.

Adzemovic and colleagues examined patients who benefited from transfer to a higher-level trauma center following initial presentation at a lower-level trauma center, and found that patients with complex solid organ injury (splenic injury grade ≥ 3), any number of rib fractures, any traumatic brain injury, pneumothorax or hemothorax, or other major fractures, had a survival benefit from transfer [23]. In this study, similarly, the main predictor of transfer was splenic injury grade ≥ 3, as well as concomitant injuries, including nearly all having a head or neck AIS ≥ 3. Contrary to our study, the aforementioned studies discuss a broader range of traumatic injuries instead of focusing on a specific injury type. Targeting a specific injury helped answer questions surrounding their management and outcomes, which may eventually assist refining transfer criteria.

Interestingly, although overall there were more males than females, significantly more females were transferred with a splenic injury grade ≥ 3 than were admitted. Female patients tended to be slightly older and have a moderately higher average ISS than males and were more commonly involved in MVCs, while males were involved predominantly in snowboarding accidents. Additionally, more females had SAE and less had operative management, while males had a higher proportion with observations and operative management, which are reflective of differences in splenic salvage upon admission to the higher level of care and admission splenic injury severity.

There was only one patient overall who failed NOM in this study. The patient was initially stable in the interventional radiology suite but went straight to surgery after blood pressure plummeted. Other studies have indicated failure rates between 6–20%, and they mostly depend on age, ISS, splenic injury grade, and appropriateness of patient selection for NOM [15, 24–29]. This patient appears to have been appropriately triaged for SAE, because the patient was young (26 years old), had an ISS of 16, a splenic injury grade of 4, bordering on 5 upon admission, and had a moderate amount of hemoperitoneum, yet was initially stable. Additionally, two patients with a splenic injury grade of 2 had surgery, because both were hemodynamically unstable upon admission, had significant chest injuries, and one was an older patient. Overall, this remote Level III trauma center appears to be appropriately and successfully triaging its splenic injury patients.

Limitations to the study include the fact that it cannot be generalized to other trauma centers with different patient populations, as it was a single, Level III trauma center in a mountainous area; however, this is one of the few studies to describe clinical characteristics of patients with splenic injury in a remote environment and their transfer characteristics. A second limitation included the small sample size after stratification by splenic injury grade was performed; however, because splenic injury grade was overly predictive of transfer status, stratifying by grade allowed us to better understand any differences in characteristics of transfer patients compared to those who were admitted.

## Conclusions

This study shows that a remote Level III trauma center is mainly transferring hemodynamically stable patients with splenic injury grade ≥ 3 and concomitant injuries, and nearly all of these patients were successfully managed nonoperatively upon arrival to a higher level of care. Stratifying blunt splenic injuries by injury grade and concomitant injuries may improve the predictability of prognosis for remote trauma centers that must expeditiously decide who must be transferred or admitted. Further studies are warranted to better delineate the potential benefits of more precise diagnostic stratification on clinical treatments and outcomes.

## Supplementary Information

**Additional file 1:** **Supplementary Table 1.**  Splenic injury clinical parameters by disposition, stratified by splenic injurygrade

**Additional file 2:** **Supplementary Table 2.**  Splenic injury management by disposition, stratified by splenic injury grade

## Data Availability

The datasets used and analyzed during the current study are available from the corresponding author on reasonable request.

## Abbreviations

OR: Odds ratio
CI: Confidence interval
CT: Computed tomography
NOM: Non-operative management
SAE: Splenic artery embolization
NOM-SAE: Non-operative management splenic artery embolization
FAST: Focused assessment with sonography
LOS: Hospital length of stay
ICU: intensive care unit
AIS: Abbreviated injury scale
ISS: Injury severity scale
COPD: Chronic obstructive pulmonary disease
MVC: Motor vehicle crash
IQR: Interquartile range
GCS: Glasgow Coma Scale
