# Supplementary material for: Predictors of transfer from a remote trauma facility to an urban level I trauma center for blunt splenic injuries: a retrospective observational multicenter study
Source: Patient Saf Surg. 2022 Sep 9;16:30. doi: 10.1186/s13037-022-00339-4 (PMC9463793; doi:10.1186/s13037-022-00339-4)
Supplement: Supplementary file 1 — Additional file 1: Supplementary Table 1. Splenic injury clinical parameters by disposition, stratified by splenic injurygrade [file 13037_2022_339_MOESM1_ESM.docx]

| Supplementary Table 1. Splenic Injury Clinical Parameters by Disposition, Stratified by Splenic Injury Grade | | | | |
| --- | --- | --- | --- | --- |
| Grade | Grades 1-2, N=30 | | Grades 3-5, N=43 | |
| Disposition | Admitted, N=24 (80%) | Transferred, N=6 (20%) | Admitted, N=11 (26%) | Transferred, N=32 (74%) |
| First Prehospital vital signs |  |  |  |  |
| *Respiration rate* | 18 (17-20) | 19 (18-20) | 18 (16-18) | 17 (16-19) |
| *Heart rate* | 83 (76.5-98.5) | 72.5 (67-90) | 88 (80-107) | 85 (70.5-99.5) |
| *Systolic blood pressure* | 124 (120-128) | 130 (120-137) | 127 (114-136) | 124 (114-133.5) |
| *Diastolic blood pressure* | 77.5 (66-80.5) | 73.5 (67-78) | 79 (66-90) | 74 (64.5-86) |
| Last pre-hospital vital signs |  |  |  |  |
| *Respiration rate* | 18 (17-20) | 20 (18-23) | 18 (16-20) | 17.5 (16-19) |
| *Heart rate* | 80 (72.5-95.5) | 87 (69-98) | 94 (90-102) | 84.5 (71.5-99.5) |
| *Systolic blood pressure* | 125 (116.5-130) | 122 (121-134) | 128 (98-136) | 127.5 (114-139.5) |
| *Diastolic blood pressure* | 73 (67.5-84.5) | 77.5 (54-79) | 76 (67-89) | 76 (66-85.5) |
| Hemoglobin arrival | 14.8 (13.7-15.5) | 14.2 (13.7-14.5) | 13.9 (11.9-15.1) | 13.8 (13.1-4.9) |
| Fast exam results |  |  |  |  |
| *Positive* | 10 (56%) | 4 (80%) | 7 (70%) | 23 (82%) |
| *Negative* | 8 (44%) | 1 (20%) | 3 (30%) | 5 (18%) |
| Hemodynamic instability prior to arrival | 2 (9%) | 0 (0%) | 2 (18%) | 1 (3%) |
| Hemodynamic instability on arrival* | 2 (9%) | 0 (0%) | 3 (27%) | 5 (16%) |
| Pseudoaneurysm | 1 (5%) | 0 (0%) | 1 (9%) | 0 (0%) |
| Contrast blush | 2 (9%) | 1 (17%) | 2 (18%) | 8 (26%) |
| Blush size |  |  |  |  |
| *Small* | 2 (100%) | 1 (100%) | 1 (50%) | 2 (25%) |
| *Moderate* | 0 (0%) | 0 (0%) | 1 (50%) | 6 (75%) |
| Hemoperitoneum | 1 (5%) | 1 (17%) | 4 (36%) | 18 (56%) |
| Hemoperitoneum size |  |  |  |  |
| *Small* | 1 (100%) | 1 (100%) | 1 (25%) | 12 (67%) |
| *Moderate* | 0 (0%) | 0 (0%) | 2 (50%) | 6 (33%) |
| *Large* | 0 (0%) | 0 (0%) | 1 (25%) | 0 (0%) |
| *Missing 1. | | | | |
|  | | | | |
