# Supplementary material for: Predictors of transfer from a remote trauma facility to an urban level I trauma center for blunt splenic injuries: a retrospective observational multicenter study
Source: Patient Saf Surg. 2022 Sep 9;16:30. doi: 10.1186/s13037-022-00339-4 (PMC9463793; doi:10.1186/s13037-022-00339-4)
Supplement: Supplementary file 2 — Additional file 2: Supplementary Table 2. Splenic injury management by disposition, stratified by splenic injury grade [file 13037_2022_339_MOESM2_ESM.docx]

| Supplementary Table 2. Splenic Injury Management by Disposition, Stratified by Splenic Injury Grade | | | | |
| --- | --- | --- | --- | --- |
| Grade | Grades 1-2, N=28 | | Grades 3-5 | |
| Disposition | Admitted, N=24 (80%) | Transferred, N=6 (20%) | Admitted, N=11 (26%) | Transferred, N=32 (74%) |
| Initial management plan |  |  |  |  |
| *NOM* | 22 (92%) | 6 (100%) | 8 (29%) | 30 (94%) |
| *OM* | 2 (8%) | 0 (0%) | 3 (27%) | 2 (6%) |
| Final management plan |  |  |  |  |
| *NOM* | 22 (92%) | 6 (100%) | 8 (29%) | 29 (91%) |
| *OM* | 2 (8%) | 0 (0%) | 3 (27%) | 3 (9%) |
| Final plan |  |  |  |  |
| *OBS* | 22 (92%) | 5 (83%) | 6 (55%) | 16 (50%) |
| *SAE* | 0 (0%) | 1 (17%) | 2 (18%) | 13 (41%) |
| *Surgery* | 2 (8%) | 0 (0%) | 3 (27%) | 3 (9%) |
| Failure of NOM | 0 (0%) | 0 (0%) | 0 (0%) | 1 (3%) |
| HLOS, median (IQR) *days* | **3 (2-3.5)** | **7.5 (5-11)** | 4 (3-7) | 5 (4-7) |
| ICU LOS, median (IQR) *days* | 3 (2-3) | 3 (3-2) | 2 (2-3) | 3 (2-5) |
| NOM, nonoperative management; OM, operative management; HLOS, hospital length of stay; ICU, intensive care unit; IQR, interquartile range. Bold numbers indicate statistical significance at p<0.05. | | | | |
|  | | | | |
